# Permeabilized microbes in biotechnology

**DOI:** 10.1111/1751-7915.12152

**Published:** 2014-08-07

**Authors:** Lawrence P Wackett

**Affiliations:** * Department of Biochemistry, Molecular Biology & Biophysics BioTechnology Institute, University of MinnesotaSt. Paul, MN, 55108, USA

## AlkL boosts biocatalytic oxygenation

### http://aem.asm.org/content/78/16/5724.full

AlkL is an outer membrane porin from *Pseudomonas*. Its expression in *E. coli* was shown to greatly stimulate the oxidation of hydrophobic substrates by a a recombinant oxygenase enzyme system.

## Enzymes for bacterial cell lysis

### http://www.sigmaaldrich.com/life-science/metabolomics/enzyme-explorer/learning-center/lysing-enzymes.html#bacterial

Bacterial membranes can be perturbed for biotechnological purposes by enzymatic treatment. Some of the enzymes and their actions are discussed on this commercial site.

## Permeabilization of *E. coli* cells to produce L-carnitine

### http://www.sciencedirect.com/science/article/pii/S0141022905000931

Different chemical treatments that are known to perturb membrane structure were used in this study to optimize the bacterial production of L-carnitine in a whole cell system.

## Chemical permeabilization of *E. coli* to release proteins

### http://deepblue.lib.umich.edu/bitstream/handle/2027.42/37899/260330712_ftp.pdf?sequence=1

Chemical permeabilization is also used to release proteins of interest from recombinant *E. coli* cells. This study focused on guanidine-HCl and Triton X100.

## Penicillin G acylase activity of permeabilized *E. coli* cells

### http://full-text.com/article/Immobilization+of+penicillin+G+acylase+using+permeabilized+Escherichia+coli+whole+cells+within+chitosan+beads,d77.html

In this study, cells were permeabilized and then immobilized to obtain a stable whole cell preparation expressing penicillin G acylase activity.

## Controlled permeabilization of *E. coli* outer membrane

### http://dissertations.ub.rug.nl/faculties/science/1989/j.p.marvin/?pLanguage=en&pFullItemRecord=ON

This thesis involved studies on outer membrane perturbation for the cellular uptake of macromolecules such as DNA.

## Microwaves produce transient membrane pores

### http://www.microbemagazine.org/index.php?option=com_content&view=article&id=3546:microwaves-can-induce-transient-pores-without-killing-e-coli-cells&catid=761&Itemid=983

An interesting approach to cell permeabilization was explored here with the use of microwaves that induce cell permeabilization with subsequent cell recovery occuring within a short time.

## Utilization of permeabilized microbial cells

### http://sdpg.pg.gda.pl/pij/files/2012/10/01_2012spec_09-Pietrow-O.-Panek-A.-Synowiecki-J.-Microbial-cells.pdf

This review discusses different methods of microbial cell permeabilization and ends with a couple specific examples.

## Bacterial membrane permeabilization: Science.gov

### http://www.science.gov/topicpages/b/bacterial+membrane+permeabilization.html

This webpage links to a compendium of papers relating to membrane permeabilization. Some are for eukaryotic membranes but many deal with prokaryotic membrane systems.

## Membrane permeabilization to obtain periplasmic protein

### https://www.microbiology.ubc.ca/sites/default/files/roles/drupal_ungrad/JEMI/14/JEMI14_1-6a.pdf

Outer membrane permeabilization of a Gram negative organism was performed to release a periplasmic protein.

## Production of D-amino acid precursors with permeabilized recombinant cells

### http://www.share-pdf.com/7708150176294b568be20d944852eef7/1-s2.0-S0032959299001570-main.htm

Recombinant hydantoinases are used to produce precursors for the synthesis of industrially-valuable D-amino acids. In this example, permeabilized cells were used as an efficient catalyst.

## Enhancement of *in vivo* activity with cetyl trimethyl ammonium bromide or acetone

### http://onlinelibrary.wiley.com/doi/10.1111/j.1472-765X.2008.02364.x/full

The production of L-phenylalanine from *trans*-cinnamic acid was enhanced by detergent and solvent treatment of an *E. coli* strain expressing a heterologous phenylalanine ammonia ligase enzyme.

## Generating porous heterogeneous catalyst analogs in *E. coli*

### http://pubs.acs.org/doi/abs/10.1021/sb400202s

In this study, the expression of a viral protein in the *E. coli* outer membrane increased the *in vivo* rate of reaction for a periplasmic protein.

## Solvent selection for whole cell biotransformations

### http://www.dbkgroup.org/Papers/salter_crc_solvents95.pdf

This review examines solvent effects for enhancing whole cell bacterial catalysts.

## Accelerating whole-cell biocatalysis by enhancing outer membrane permeability: A thesis

### https://digarchive.library.vcu.edu/bitstream/handle/10156/1679/niy_phd.pdf?sequence=1

This thesis research investigated the modification of *E. coli* outer membrane lipoprotein to enhance entrance of chemicals into the cell to carry out biotransformations.

## Cetyl-trimethylammonium bromide pretreatment for enhanced synthesis of a flavor ester

### http://www.plosone.org/article/info%3Adoi%2F10.1371%2Fjournal.pone.0091872

This lipase-dependent *E. coli* whole cell biocatalyst system used a cetyl-trimethylammonium bromide pretreament to obtain a six-fold higher synthetic activity.

